# Effects of upadacitinib on patient-reported outcomes: results from SELECT-BEYOND, a phase 3 randomized trial in patients with rheumatoid arthritis and inadequate responses to biologic disease-modifying antirheumatic drugs

**DOI:** 10.1186/s13075-019-2059-8

**Published:** 2019-12-02

**Authors:** Vibeke Strand, Michael Schiff, Namita Tundia, Alan Friedman, Sebastian Meerwein, Aileen Pangan, Arijit Ganguli, Mahesh Fuldeore, Yan Song, Janet Pope

**Affiliations:** 1 Stanford University, 306 Ramona Road, Portola Valley, CA 94028 USA; 2 University of Colorado School of Medicine, Denver, CO 80045 USA; 3 AbbVie Inc., 1 North Waukegan Road, North Chicago, IL 60064 USA; 4 AbbVie Deutschland GmbH & Co., KG, Mainzer Strasse 81, 65189 Wiesbaden, Germany; 5 Analysis Group Inc., 14th Floor, 111 Huntington Avenue, Boston, MA 02199 USA; 6 University of Western Ontario, St. Joseph’s Health Care, 268 Grosvenor Street, London, ON N6A 4V2 Canada

**Keywords:** Rheumatoid arthritis, Patient-reported outcome measures, JAK inhibitor, Quality of life, Treatment outcomes, Upadacitinib, Fatigue, HAQ, Pain, SF-36, MCID

## Abstract

**Background:**

Patient-reported outcomes (PROs) are important when evaluating treatment benefits in rheumatoid arthritis (RA). We compared upadacitinib, an oral, selective JAK-1 inhibitor, with placebo to assess clinically meaningful improvements in PROs in patients with RA who have had inadequate responses to biologic disease-modifying antirheumatic drugs (bDMARD-IR).

**Methods:**

PRO responses between upadacitinib 15 mg or 30 mg and placebo were evaluated at week 12 from the SELECT-BEYOND trial. Improvement was determined by measuring Patient Global Assessment of Disease Activity (PtGA), pain, Health Assessment Questionnaire Disability Index (HAQ-DI), Short Form-36 Health Survey (SF-36), duration and severity of morning (AM) stiffness, and Insomnia Severity Index (ISI). Least squares mean changes and percentage of patients reporting improvements ≥ minimum clinically important differences (MCID) and scores greater than or equal to normative values were determined. The number needed to treat (NNT) to achieve clinically meaningful improvements was calculated.

**Results:**

In 498 patients, both upadacitinib doses resulted in statistically significant changes from baseline versus placebo in PtGA, pain, HAQ-DI, SF-36 Physical Component Summary (PCS), 7 of 8 SF-36 domains (15 mg), 6 of 8 SF-36 domains (30 mg), and AM stiffness duration and severity. Compared with placebo, more upadacitinib-treated patients reported improvements ≥ MCID in PtGA, pain, HAQ-DI, SF-36 PCS, 7 of 8 SF-36 domains (15 mg), 5 of 8 SF-36 domains (30 mg), AM stiffness duration and severity, and ISI (30 mg) and scores ≥ normative values in HAQ-DI and SF-36 domains. Across most PROs, NNTs to achieve MCID with upadacitinib ranged from 4 to 7 patients.

**Conclusions:**

In bDMARD-IR RA patients, upadacitinib (15 mg or 30 mg) improved multiple aspects of quality of life, and more patients reached clinically meaningful improvements approaching normative values compared with placebo.

**Trial registration:**

The trial is registered with ClinicalTrials.gov (NCT02706847), registered 6 March 2016.

## Background

Rheumatoid arthritis (RA) is a chronic, inflammatory, and destructive disease of the synovial joints that is associated with substantial clinical burden, reduced health-related quality of life (HRQOL), and shortened life expectancy [1, 2]. Most patients with RA experience impaired physical functioning, chronic pain, fatigue, and morning (AM) stiffness, which affect their HRQOL [2–8]. In addition, patients with RA have reported that sleep disturbances are a key determinant of their well-being [3, 9–11]. These reports are supported by cross-sectional studies, which have demonstrated that sleep disturbance correlates with greater pain, disease activity, and fatigue in RA [12–15]. Thus, understanding the patient’s perspective of how a therapy impacts multiple aspects of HRQOL is crucial when evaluating the efficacy of treatments for RA [16–18].

Despite several treatment options, a significant proportion of patients with RA become refractory, or do not adequately respond, to available therapies [19–21]. Several classes of drugs are available to treat inflammation and thereby provide relief from symptoms associated with RA. Current treatment options include conventional synthetic disease-modifying antirheumatic drugs (csDMARDs) such as methotrexate (MTX), biologic DMARDs (bDMARDs), and targeted synthetic DMARDs (tsDMARDs) [2, 22]. Janus kinase (JAK) inhibitors are a new class of tsDMARDs approved for use in RA [2, 22]. Upadacitinib, a potent JAK inhibitor with preferential activity against JAK1, has been evaluated across RA populations (MTX-naive, MTX-inadequate responders [MTX-IR], csDMARD-inadequate responders [csDMARD-IR], and bDMARD-inadequate responders [bDMARD-IR]) both as monotherapy and in combination with csDMARDs [23–26].

SELECT-BEYOND is a phase 3, randomized controlled trial (RCT) of upadacitinib 15 mg or 30 mg once daily in patients with RA who are bDMARD-IR and receiving background csDMARDs. The RCT demonstrated that significantly more upadacitinib-treated patients had American College of Rheumatology 20% improvement (ACR20) responses and lower disease activity than placebo [25]. The objectives of the following analyses are to compare response rates and numbers needed to treat (NNTs) with upadacitinib versus placebo for PROs and assess the achievement of clinically meaningful improvements and normative values in patients with treatment-refractory RA.

## Methods

### Study design and participants

The full study design of SELECT-BEYOND, a phase 3 RCT (NCT02706847), has been published previously [24, 25]. Patients aged 18 years and older with moderate to severe RA for ≥ 3 months were randomized 1:1:1 to receive oral upadacitinib 15 or 30 mg once daily, or placebo, for 12 weeks. Patients were excluded if they had prior exposure to a JAK inhibitor. The study was approved by independent ethics committees or institutional review boards at each study site and conducted in accordance with ethical principles outlined in the current Declaration of Helsinki and consistent with International Conference on Harmonisation Good Clinical Practice and Good Epidemiology Practices, along with all applicable local regulatory requirements. All patient data were deidentified and complied with patient confidentiality requirements.

### Patient-reported outcome assessment

Several clinically relevant PROs and assessments were used to evaluate the potential impact of upadacitinib on patient HRQOL and disease burden. These include Patient Global Assessment of Disease Activity (PtGA); pain on a visual analog scale (VAS); Health Assessment Questionnaire Disability Index (HAQ-DI) [27]; HRQOL using the Short Form 36 Health Survey (SF-36), which includes 8 domains scored 0–100 (physical functioning, role-physical, bodily pain, general health, vitality, social functioning, role-emotional, and mental health) and 2 aggregate Physical (PCS) and Mental Component Summary (MCS) scores (range of 0–100) [28, 29] with higher scores indicating better HRQOL; AM stiffness severity measured by a numeric rating scale ranging from 0 to 10 with higher scores indicating greater severity, duration of AM stiffness measured in minutes [8, 30, 31]; the Insomnia Severity Index (ISI) to identify and grade insomnia severity (scores range from 0 to 28 where higher scores indicate increased insomnia) [32–34]; and the Euro Qol 5-Dimension 5-Level Questionnaire (EQ-5D-5L), which includes a general health status index measured by a 0–100 VAS and consists of 5 health states [35].

Clinically meaningful responses for each PRO were defined as changes from baseline greater than or equal to minimal clinically important difference (MCID) or greater than or equal to normative values. MCIDs were defined as ≥ 10-point decreases for PtGA [30], ≥ 10-point decreases for pain VAS [30], ≥ 0.22-point decreases for HAQ-DI [30], ≥ 2.5-point increases in SF-36 PCS and MCS [36], ≥ 5-point increases in SF-36 domains [36], ≥ 8.4-point decreases for ISI (moderate improvement) [32], and ≥ 0.05-cm increases for EQ-5D-5L [35]. Owing to the lack of a predefined MCID for AM stiffness in the literature, the minimum important difference (MID) was defined as a reduction of ≥ 1 point for severity and one half standard deviation of the mean baseline values for duration of AM stiffness [8, 30, 31]. Normative values for PROs were obtained from the literature and defined as the following: ≤ 20 for PtGA [37], 0.25 for HAQ-DI [38], 50 for SF-36 PCS and MCS [39], 0–7 for ISI [33], and 0.915 for EQ-5D-5L [40]. Normative values are not available for pain VAS or AM stiffness [8, 30, 31, 41]. The eight SF-36 domains were compared with age- and gender-matched (A/G) normative US population values as a benchmark [42].

### Statistical analyses

All patients included in the intention-to-treat population of the BEYOND RCT were eligible for this post hoc analysis. Mean changes were calculated as changes in least squares mean (LSM) from baseline to week 12 for upadacitinib versus placebo based on a mixed effect for repeated measures model. Responses for each PRO were estimated at weeks 1, 4, and 12. Non-responder imputation (NRI) was used to impute missing responses. Between-group differences in responses were assessed using chi-square tests. Time to response was assessed by the Kaplan-Meier analysis and was compared using log-rank test. “Spydergrams” were used to compare SF-36 domain responses [42]. In this study, NNTs were measured as the number of patients needed to achieve one additional responder, defined as the reciprocal of the response rate difference between treatment and placebo groups, and were calculated for each PRO at week 12.

## Results

**Fig. 1:**
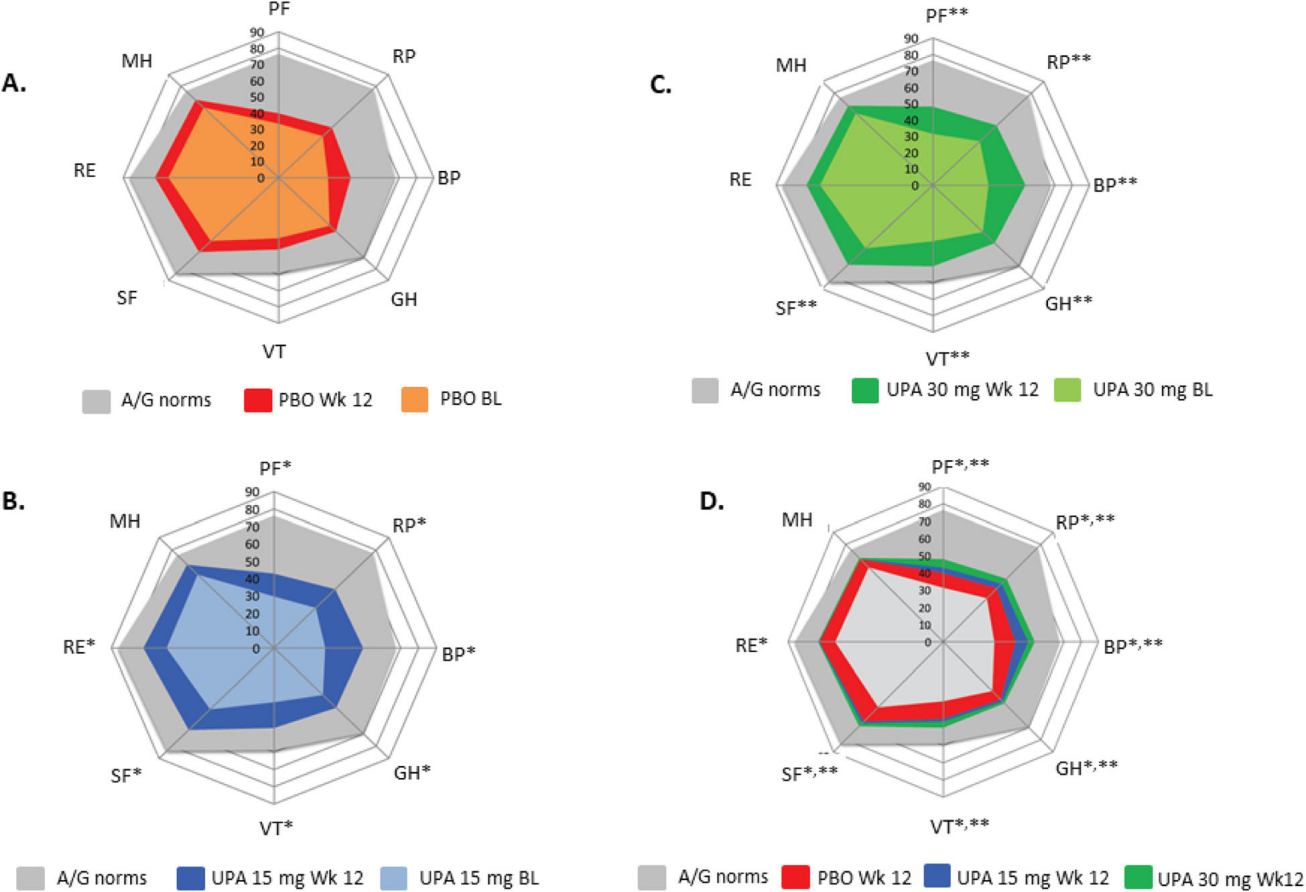
BL and week 12 scores across SF-36 domains relative to age- and gender-adjusted (A/G) norms for the general US population. **a** Placebo. **b** UPA 15 mg. **c** UPA 30 mg. **d** Combined. All scores were based on a scale of 0 to 100, where 0 is the worst and 100 is the best. No further transformations were applied. *P < 0.05 for UPA 15 mg vs PBO. **P < 0.05 for UPA 30 mg vs PBO. BL, baseline; BP, bodily pain; GH, general health; MH, mental health; PBO, placebo; PF, physical functioning; RE, role-emotional; RP, role-physical; SF, social functioning; SF-36, Short Form-36 Health Survey; UPA, upadacitinib; VT, vitality; Wk, week

Patient disposition and demographic information have been published [24]. Disease characteristics and baseline PRO values across treatment groups were well-balanced (Tables 1 and 2). Decrements in PROs from normative values at baseline indicate that patients in this RCT reported substantial impairments in HRQOL. SF-36 mean baseline domain scores were approximately 25–50 points lower than in the A/G normative US population (Fig. 1).

**Table 1:** Patient demographics and baseline characteristics

Characteristic	Placebo (*n* = 169)	Upadacitinib 15 mg (*n* = 164)	Upadacitinib 30 mg (*n* = 165)
Age (y), mean ± SD	57.6 ± 11.4	56.3 ± 11.3	57.3 ± 11.6
Female, *n* (%)	143 (84.6)	137 (83.5)	138 (83.6)
Race, *n* (%)			
White	143 (84.6)	142 (86.6)	148 (89.7)
Black	21 (12.4)	17 (10.4)	10 (6.1)
Asian	5 (3.0)	2 (1.2)	2 (1.2)
Other	0 (0)	3 (1.8)	5 (3.0)
BMI (kg/m^2^), mean ± SD	29.7 ± 7.4	31.2 ± 7.3	29.7 ± 6.2
Duration of RA (y), mean ± SD	14.5 ± 9.2	12.4 ± 9.4	12.7 ± 9.7
Failed ≥ 1 anti-TNF, *n* (%)	152 (89.9)	146 (89.0)	151 (92.1)
Failed ≥ 1 bDMARD due to lack of efficacy, *n* (%)	159 (94.1)	146 (89.0)	139 (84.8)

*bDMARD* biologic disease-modifying antirheumatic drug, *RA* rheumatoid arthritis, *SD* standard deviation, *TNF* tumor necrosis factor, *y* years

**Table 2:** Baseline values and LSM changes from baseline at week 12

PRO	Baseline value, mean ± SD			LSM change from baseline (95% CI)		
	Placebo	Upadacitinib 15 mg	Upadacitinib 30 mg	Placebo	Upadacitinib 15 mg	Upadacitinib 30 mg
PtGA	66.3 ± 22.7 (*n* = 166)	67.2 ± 19.6 (*n* = 163)	64.7 ± 21.1 (*n* = 163)	− 10.03 (− 14.22, − 5.84) (*n* = 145)	− 26.04* (− 30.16, − 21.93) (*n* = 156)	− 29.27* (− 33.43, − 25.12) (*n* = 147)
Pain VAS	68.9 ± 21.0 (*n* = 166)	68.2 ± 19.8 (*n* = 163)	65.3 ± 20.7 (*n* = 161)	− 10.38 (− 14.60, − 6.16) (*n* = 145)	− 25.91* (− 30.05, − 21.76) (*n* = 156)	− 30.92* (− 35.12, − 26.72) (*n* = 146)
HAQ-DI	1.6 ± 0.6 (*n* = 166)	1.7 ± 0.6 (*n* = 163)	1.6 ± 0.6 (*n* = 161)	− 0.16 (− 0.25, − 0.08) (*n* = 145)	− 0.41* (− 0.50, − 0.33) (*n* = 156)	− 0.44* (− 0.52, − 0.35) (*n* = 146)
SF-36 PCS	31.6 ± 7.2 (*n* = 166)	30.6 ± 7.8 (*n* = 163)	31.5 ± 7.3 (*n* = 162)	2.39 (1.14, 3.64) (*n* = 145)	5.83* (4.60, 7.05) (*n* = 156)	7.02* (5.78, 8.25) (*n* = 147)
SF-36 MCS	45.9 ± 12.6 (*n* = 166)	44.0 ± 11.7 (*n* = 163)	45.9 ± 12.3 (*n* = 162)	3.01 (1.65, 4.37) (*n* = 145)	4.54 (3.22, 5.87) (*n* = 156)	3.37 (2.03, 4.72) (*n* = 147)
SF-36 PF	32.0 ± 8.9 (*n* = 166)	30.6 ± 9.3 (*n* = 163)	31.2 ± 8.1 (*n* = 162)	1.54 (0.25, 2.84) (*n* = 145)	4.56* (3.29, 5.83) (*n* = 156)	6.15* (4.87, 7.43) (*n* = 147)
SF-36 RP	34.2 ± 8.0 (*n* = 166)	33.0 ± 8.8 (*n* = 163)	34.9 ± 7.6 (*n* = 162)	2.15 (0.95, 3.35) (*n* = 145)	5.10* (3.93, 6.27) (*n* = 156)	5.17* (3.99, 6.36) (*n* = 147)
SF-36 BP	33.2 (6.4) (*n* = 166)	33.1 (6.4) (*n* = 163)	34.5 (6.9) (*n* = 162)	4.71 (3.38, 6.04) (*n* = 145)	8.12* (6.82, 9.42) (*n* = 156)	9.09* (7.78, 10.41) (*n* = 147)
SF-36 GH	38.9 ± 9.7 (*n* = 166)	37.1 ± 8.2 (*n* = 163)	38.1 ± 9.1 (*n* = 162)	2.17 (1.01, 3.34) (*n* = 145)	4.31** (3.17, 5.45) (*n* = 156)	4.25*** (3.10, 5.41) (*n* = 147)
SF-36 VT	40.6 ± 9.3 (*n* = 166)	37.7 ± 10.0 (*n* = 163)	39.2 ± 9.4 (*n* = 162)	3.10 (1.69, 4.51) (*n* = 145)	6.28* (4.90, 7.65) (*n* = 156)	6.86* (5.47, 8.26) (*n* = 147)
SF-36 SF	39.5 ± 10.9 (*n* = 166)	37.3 ± 10.4 (*n* = 163)	39.1 ± 10.8 (*n* = 162)	3.45 (2.10, 4.81) (*n* = 145)	5.87*** (4.55, 7.19) (*n* = 156)	5.35*** (4.01, 6.69) (*n* = 147)
SF-36 RE	41.0 ± 12.6 (*n* = 166)	39.2 ± 12.7 (*n* = 163)	41.5 ± 12.8 (*n* = 162)	2.66 (1.24, 4.07) (*n* = 145)	4.72*** (3.34, 6.10) (*n* = 156)	3.78 (2.38, 5.17) (*n* = 147)
SF-36 MH	43.6 ± 12.5 (*n* = 166)	42.9 ± 11.3 (*n* = 163)	44.2 ± 11.4 (*n* = 162)	2.96 (1.63, 4.29) (*n* = 145)	3.83 (2.53, 5.12) (*n* = 156)	3.53 (2.21, 4.84) (*n* = 147)
AM stiffness duration, minutes	138.4 ± 178.6 (*n* = 169)	140.4 ± 189.7 (*n* = 164)	184.5 ± 284.9 (*n* = 165)	− 15.07 (− 43.30, 13.16) (*n* = 147)	− 81.47* (− 109.52, − 53.42) (*n* = 157)	− 79.13* (− 107.26, − 51.00) (*n* = 148)
AM stiffness severity	6.8 ± 2.3 (*n* = 169)	6.8 ± 2.1 (*n* = 164)	6.5 ± 2.2 (*n* = 165)	− 1.57 (− 1.98, − 1.17) (*n* = 146)	− 2.86* (− 3.26, − 2.46) (*n* = 157)	− 3.22* (− 3.62, − 2.82) (*n* = 147)
ISI	12.3 ± 6.9 (*n* = 153)	12.3 ± 6.9 (*n* = 152)	12.3 ± 6.6 (*n* = 151)	− 1.69 (− 2.55, − 0.83) (*n* = 130)	− 2.53 (− 3.36, − 1.70) (*n* = 142)	− 3.32** (− 4.15, − 2.49) (*n* = 138)
EQ-5D-5L	49.7 ± 24.9 (*n* = 166)	50.7 ± 23.0 (*n* = 163)	51.8 ± 21.8 (*n* = 160)	7.45 (3.86, 11.04) (*n* = 145)	15.03** (11.55, 18.51) (*n* = 156)	15.12** (11.55, 18.69) (*n* = 145)

*AM* morning, *BP* bodily pain, *CI* confidence interval, *EQ-5D-5L* Euro Qol 5-Dimension 5-Level Questionnaire, *GH* general health, *HAQ-DI* Health Assessment Questionnaire Disability Index, *LSM* least squares mean, *MCS* Mental Component Summary, *MH* mental health, *PCS* Physical Component Summary, *PF* physical functioning, *PRO* patient-reported outcome, *PtGA* Patient Global Assessment of Disease Activity, *RE* role-emotional, *RP* role-physical, *SD* standard deviation, *SF* social functioning, *SF-36* Short Form-36 Health Survey, *VAS* visual analog scale, *VT* vitality

*P ≤ 0.001 for upadacitinib vs placebo

**P < 0.01 for upadacitinib vs placebo

***P < 0.05 for upadacitinib vs placebo

**Fig. 2:**
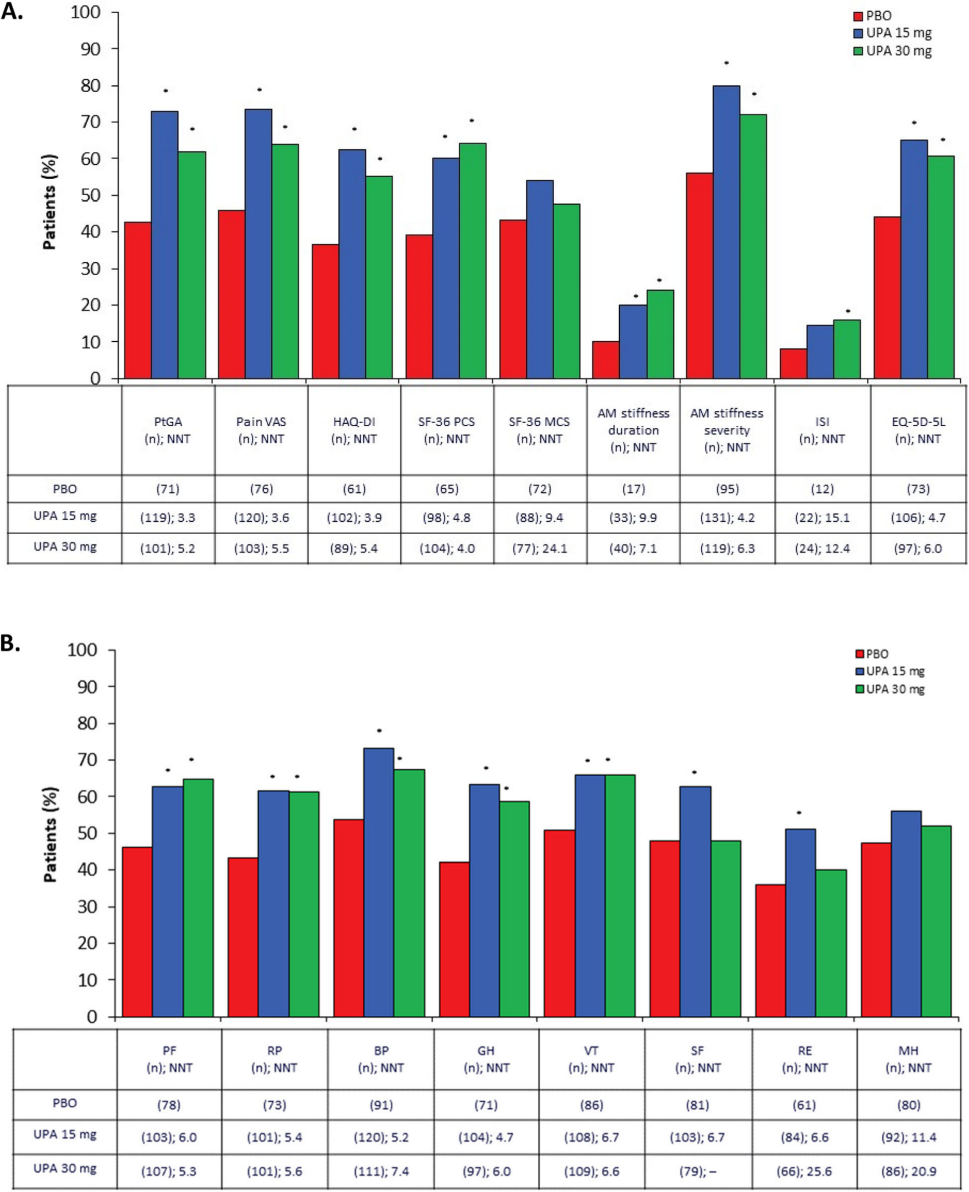
Patients reporting improvements ≥ MCID at week 12 across PROs. **a** Results from multiple patient health-related quality of life assessments. **b** Results from the SF-36 subdomains. SF-36 domains were rescored from 0 to 100, where 0 is the worst and 100 is the best. No further transformations were applied. *P < 0.05 for UPA vs PBO. AM, morning; BP, bodily pain; EQ-5D-5L, Euro Qol 5-Dimension 5-Level Questionnaire; GH, general health; HAQ-DI, Health Assessment Questionnaire Disability Index; ISI, Insomnia Severity Index; MCID, minimum clinically important difference; MCS, Mental Component Summary; MH, mental health; NNT, number needed to treat; PBO, placebo; PCS, Physical Component Summary; PF, physical functioning; PRO, patient-reported outcome; PtGA, Patient Global Assessment of Disease Activity; RE, role-emotional; RP, role-physical; SF, social functioning; SF-36, Short Form-36 Health Survey; UPA, upadacitinib; VAS, visual analog scale; VT, vitality

Compared with placebo, statistically significant improvements at week 12 were evident with both upadacitinib 15 mg and 30 mg for PtGA, pain VAS, HAQ-DI, PCS, and AM stiffness (all *P* ≤ 0.001, Table 2). Duration of AM stiffness was reduced from baseline by 43% and 58% in the upadacitinib 15 mg and 30 mg groups, respectively, versus 11% in the placebo group, and 72% and 80% of patients receiving upadacitinib 15 mg and 30 mg, respectively, versus 52% of patients receiving placebo reported a reduction in severity greater than or equal to MID. MCS baseline values were close to normative values, and although changes from baseline were numerically greater with upadacitinib 15 mg and 30 mg (4.54 and 3.37, respectively) compared with placebo (3.01), they were not statistically significant (*P* = 0.52). Changes from baseline in SF-36 domain scores with upadacitinib 15 mg exceeded placebo across all eight domains and were statistically significant except in the SF-36 mental health domain. For upadacitinib 30 mg, changes from baseline were statistically significant across all domains except mental health and role-emotional. All mean improvements for upadacitinib were clinically meaningful (Fig. 2). NNTs for upadacitinib 15 mg versus placebo ranged from 3 to 4 for PtGA, pain VAS, and HAQ-DI; 4 to 5 for PCS and AM stiffness severity; and 5 to 7 for seven of eight SF-36 domains. Similar results were reported with upadacitinib 30 mg.

**Fig. 3:**
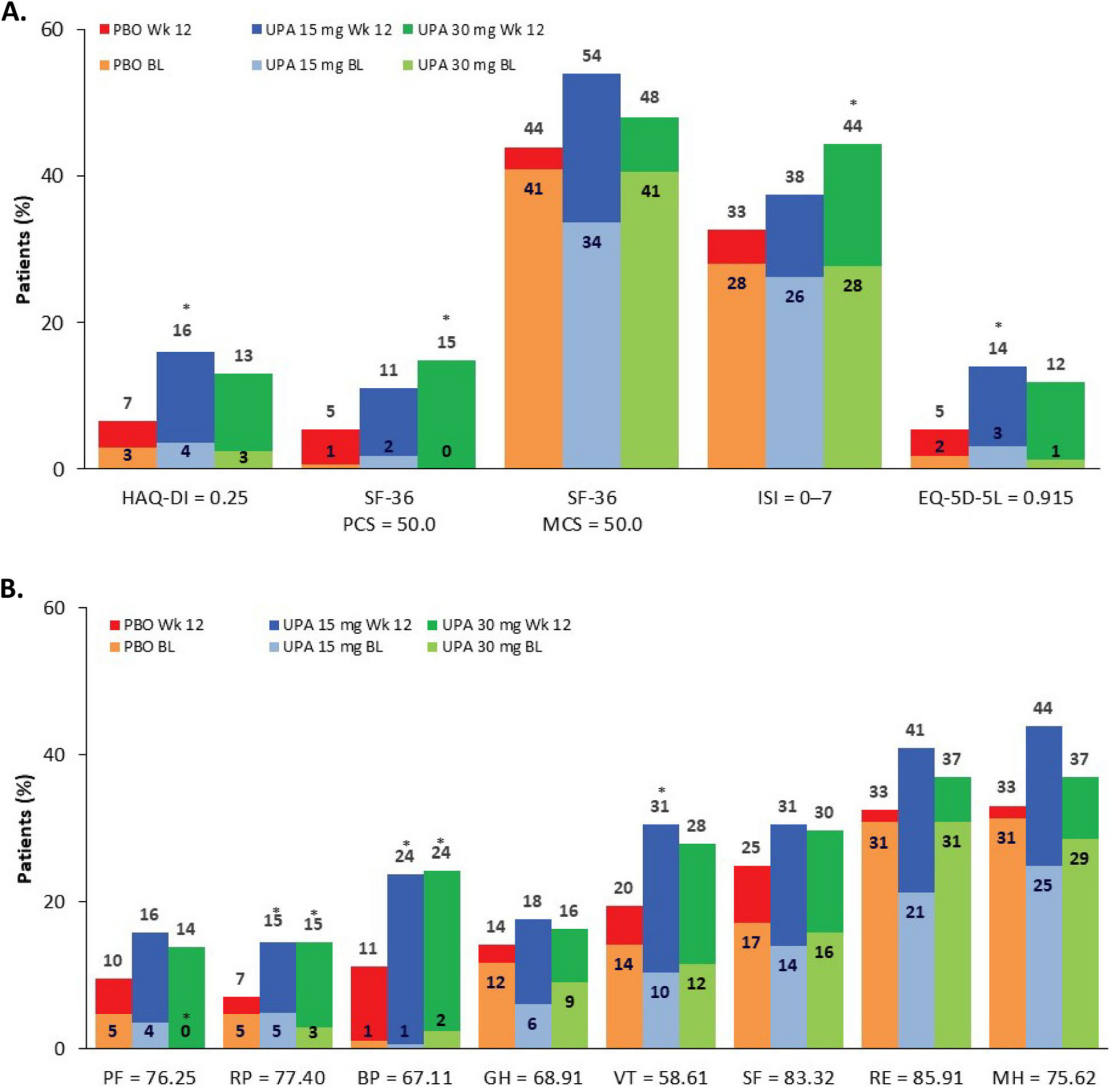
Patients reporting scores ≥ normative values. **a** Select PROs at baseline and week 12. **b** Short Form 36 (SF-36) domains. SF-36 domains were rescored from 0 to 100, where 0 is the worst and 100 is the best. No further transformations were applied. *P < 0.05 for UPA vs PBO. BL, baseline; BP, bodily pain; EQ-5D-5L, Euro Qol 5-Dimension 5-Level Questionnaire; GH, general health; HAQ-DI, Health Assessment Questionnaire Disability Index; ISI, Insomnia Severity Index; MCS, Mental Component Summary; MH, mental health; PBO, placebo; PCS, Physical Component Summary; PF, physical functioning; PRO, patient-reported outcome; RE, role-emotional; RP, role-physical; SF, social functioning; SF-36, Short Form-36 Health Survey; UPA, upadacitinib; VT, vitality; Wk, week

In both upadacitinib groups, median time to response was 2 weeks for pain VAS and HAQ-DI compared with 4 weeks for these scores in the placebo group. Time to response for severity of AM stiffness was 1 week for both upadacitinib cohorts versus 2 weeks for the placebo cohort. At week 12, a significantly greater percentage of patients in the upadacitinib 15 mg group versus the placebo group reported scores greater than or equal to normative values in PtGA (28% vs 15%); HAQ-DI (16% vs 7%); the role-physical (15% vs 7%), bodily pain (24% vs 11%), and vitality (31% vs 20%) domains; and EQ-5D-5L (14% vs 5%) (all *P* < 0.05). Likewise, upadacitinib 30 mg had significantly more patients report scores greater than or equal to normative values compared with placebo in PtGA (36% vs 15%), PCS (15% vs 5%), role-physical (15% vs 7%) and bodily pain (24% vs 11%) domains, and ISI (44% vs 33%) (all *P* < 0.05, Fig. 3).

## Discussion

Although there are a number of first-line therapies that improve RA outcomes, these same therapies may not be efficacious as second- or third-line therapy [19]. Additionally, not only is it essential to demonstrate that new treatments reduce the signs and symptoms of active RA in patients with refractory disease, but it is also necessary to show that these new treatments improve HRQOL outcomes from the patient’s perspective. We analyzed data collected on several measures during SELECT-BEYOND to gain insight into the benefits of upadacitinib on HRQOL in patients with treatment-refractory RA. Upadacitinib treatment resulted in rapid and clinically meaningful improvements in outcomes of importance to patients with refractory disease: disease activity, pain, physical function, and AM stiffness, even in a difficult to treat population. Upadacitinib 15 mg and 30 mg had an equally effective impact on most of the PROs in this bDMARD-IR RA population. Upadacitinib-treated patients reported a substantial reduction in duration of AM stiffness and a significant decrease in severity as early as week 1. Reduced sleep quality is common among patients with RA and is associated with disease activity, pain, and functional disability [43, 44]. Consistent with this finding, upadacitinib-treated patients with improvements in PtGA, pain VAS, and HAQ-DI scores also reported significant improvement in ISI scores. These clinically relevant improvements in PROs are consistent with the positive efficacy findings reported in this csDMARD-IR population [25, 45] and suggest that upadacitinib may be an important treatment option in patients with active and refractory RA.

Although a number of studies have shown that patients with RA who fail to respond to one anti-TNF agent may benefit from treatment with a second or third anti-TNF agent [46–51], the response is likely to decline as the number of anti-TNF agents increases [52]. Less information is available for the more recently approved JAK inhibitors and interleukin-6 receptor antagonist. In ORAL-STEP [53], a phase 3 RCT of tofacitinib in bDMARD-IR patients, the percentage of tofacitinib-treated (5 mg) patients reporting improvements ≥ MCID was less than in upadacitinib-treated (15 mg) patients in PtGA (65% vs 73%) and pain (69% vs 74%), similar in HAQ-DI (61% vs 63%) and SF-36 MCS (54% each), and greater in SF-36 PCS (68% vs 60%). Different results were observed in RA-BEACON [54], a phase 3 RCT of baricitinib in bDMARD-IR patients, where fewer baricitinib-treated (2 mg) patients reported improvements ≥ MCID than those treated with upadacitinib (15 mg) in SF-36 PCS (49% vs 60%) and MCS (33% vs 54%). In TARGET [55], a phase 3 RCT of sarilumab in bDMARD-IR patients, fewer sarilumab-treated (200 mg) patients reported improvements ≥ MCID than those treated with upadacitinib in PtGA (25% vs 73%), pain (26% vs 74%), HAQ-DI (21% vs 63%), SF-36 PCS (21% vs 60%), and SF-36 MCS (17% vs 54%). Treatment with upadacitinib, baricitinib [54], or sarilumab [55] significantly improved AM stiffness compared with placebo; however, it is difficult to compare results across studies because AM stiffness was assessed differently in these studies. BEYOND reported mean change in duration, RA-Beacon reported median change in duration, and TARGET reported change on a VAS scale. Overall, upadacitinib showed improvements in PROs that were better or comparable to those seen with the IL-6 receptor antagonist or other JAK inhibitors [53–55].

There are several strengths in these analyses. Data were collected during a phase 3 RCT, which ensures patients are closely followed for an extended period and PROs are consistently measured. The randomized and blinded study design mitigates biases that may arise due to differences between treatment groups without knowledge of treatment allocation. The blinded study design also allows for unbiased reporting from a patient’s perspective. The validated PROs used in these analyses evaluate different aspects of the patient’s experience which may shed light on how patients perceive the effects of upadacitinib on a wide range of typical RA-related impediments. Use of MCID and normative values to measure responses makes these data clinically meaningful and interpretable for patients.

There are also limitations in these results. PROs were collected at fixed visits, and therefore, response could not be assessed at time intervals between visits. Because the duration of treatment was relatively short (12 weeks), additional studies are needed to determine if the improvements observed with upadacitinib treatment are maintained long-term. Furthermore, results may not be generalizable to all patients with RA, as clinical trial participants are selected based on specific inclusion/exclusion criteria and may differ from the broader RA patient population in clinical practice. The patients were bDMARD treatment failures, and results could be different in other scenarios. Lastly, the method used to impute missing data (NRI) assumes that missing PRO scores are associated with non-response, which may underestimate the true rate of response.

## Conclusion

In conclusion, over 12 weeks, results from the SELECT-BEYOND trial demonstrated that in difficult-to-treat bDMARD-IR patients with active RA, treatment with upadacitinib compared with placebo resulted in significantly more patients with clinically meaningful improvements in PROs and responses that approached normative values. Furthermore, the NNTs to achieve clinically meaningful responses were ≤ 10, which are generally considered favorable [56]. Upadacitinib may be a treatment option for bDMARD-IR patients with RA providing clinically significant relief from symptoms that impair HRQOL.

## Data Availability

The datasets used and/or analyzed during the current study are available from the corresponding author on reasonable request.

## Abbreviations

ACR20: American College of Rheumatology 20% improvement
A/G: Age- and gender-matched
AM: Morning
bDMARD: Biologic disease-modifying antirheumatic drug
csDMARD: Conventional synthetic disease modifying antirheumatic drug
EQ-5D-5L: Euro Qol 5-Dimension 5-Level Questionnaire
HAQ-DI: Health Assessment Questionnaire Disability Index
HRQOL: Health-related quality of life
IR: Inadequate responder
ISI: Insomnia Severity Index
JAK: Janus kinase
LSM: Least squares mean
MCID: Minimum clinically important differences
MCS: Mental Component Summary
MTX: Methotrexate
NNT: Number needed to treat
NRI: Non-responder imputation
PCS: Physical Component Summary
PRO: Patient-reported outcome
PtGA: Patient Global Assessment of Disease Activity
RA: Rheumatoid arthritis
RCT: Randomized controlled trial
SF-6D: Short-Form Six Dimension
SF-36: Short Form-36 Health Survey
tsDMARD: Targeted synthetic biologic disease-modifying antirheumatic drug
VAS: Visual analog scale
